# A genetic bistable switch utilizing nonlinear protein degradation

**DOI:** 10.1186/1754-1611-6-9

**Published:** 2012-07-09

**Authors:** Daniel Huang, William J Holtz, Michel M Maharbiz

**Affiliations:** 1 Department of Electrical Engineering and Computer Science, University of California, 656 Sutardja Dai Hall,Berkeley, Berkeley, CA, 94720, USA; 2 California Institute for Quantitative Biosciences, University of California Berkeley, 5885 Hollis St, Emeryville, CA, 94608, USA

## Abstract

**Background:**

Bistability is a fundamental property in engineered and natural systems, conferring the ability to switch and retain states. Synthetic bistable switches in prokaryotes have mainly utilized transcriptional components in their construction. Using both transcriptional and enzymatic components, creating a hybrid system, allows for wider bistable parameter ranges in a circuit.

**Results:**

In this paper, we demonstrate a tunable family of hybrid bistable switches in *E. coli* using both transcriptional components and an enzymatic component. The design contains two linked positive feedback loops. The first loop utilizes the lambda repressor, CI, and the second positive feedback loop incorporates the Lon protease found in *Mesoplasma florum* (*mf*-Lon). We experimentally tested for bistable behavior in exponential growth phase, and found that our hybrid bistable switch was able to retain its state in the absence of an input signal throughout 40 cycles of cell division. We also tested the transient behavior of our switch and found that switching speeds can be tuned by changing the expression rate of *mf*-Lon.

**Conclusions:**

To our knowledge, this work demonstrates the first use of dynamic expression of an orthogonal and heterologous protease to tune a nonlinear protein degradation circuit. The hybrid switch is potentially a more robust and tunable topology for use in prokaryotic systems.

## Introduction

As is well known, bistability is a fundamental property of a vast array of engineered and natural systems. Bistability confers the ability to switch, to retain state, and to store information, all three comprising a closely related set of operations common to computational systems. Not surprisingly, bistable networks and components are found to be central to many cellular processes, ranging from cell cycle and differentiation, to cell fate determinations and environmental sensing [1-6]. Over the last decade, a great deal of interest has arisen towards the creation of synthetic bistable genetic systems for use as memories, decision making circuits or sustained response devices [7-9]. Many studies have focused on understanding and improving these systems, and creating proper design rules for building synthetic bistable switches and modifying existing systems [10-13].

The most basic bistable switch is a single positive feedback loop. Bistable switches consisting of a single positive feedback loop are rarely observed in nature. The first synthetic bistable genetic switch was a single positive feedback loop constructed with a pair of co-antagonistic repressors [14]. Even in the absence of cooperativity, a single positive feedback loop containing a transcriptional activator and its cognate promoter can exhibit bistable behavior due to transcriptional nonlinearity from cell growth [15]; employing variations resulting from cell growth, however, has proven difficult to exploit. Both natural and synthetic systems often contain multiple feedback loops for added control and robustness [10,11,16].

Protein degradation rates have also been mentioned to be critical in the operation of bistable and bimodal networks [17]; bimodal gene expression can result simply from changing protein degradation rates [18,19]. The *Vibrio fisheri* quorum sensing system is an example from nature that uses nonlinear degradation to create a bistable system. Here nonlinear degradation is achieved through cooperative stability, where proteins are protected against degradation by protein multimerization. The positive transcription factor LuxR turns on the *lux* promoter, producing more LuxR. LuxR as a monomer is unstable and has a fast degradation rate. The LuxR monomer does not accumulate to high concentrations and also cannot activate the *lux* promoter. In the presence of the autoinducer AHL, LuxR dimerizes into a stable and slow-degrading complex. The LuxR-AHL complex is highly stable, is able to accumulate to a high concentration and is also able to activate the *lux* promoter. Varying the levels of AHL can switch the *lux* promoter between “on” and “off” states. Unfortunately, creating a synthetic bistable system utilizing cooperative stability is difficult, requiring protein engineering to create synthetic proteins with cooperative stability.

Nonlinear degradation conditions can also be achieved through dynamic protein degradation. Dynamic protein degradation has been observed in *Xenopus*, and is responsible for cell cycle progression. The concentration of Cdc2-cyclin B increases during interphase, but abruptly drops during mitosis. The accumulation of Cdc2-cyclin B actives the anaphase promoting complex (APC), and the APC inactivates Cdc2-cyclin B through proteolysis. A mitotic oscillator is observed in *Xenopus* during the early embryonic cell cycle [20], by taking advantage of dynamic degradation of Cdc2-cyclin B.

All reported synthetic bistable switches implemented in prokaryotes exclusively use transcriptional components (i.e. transcriptional activators, repressors and their cognate promoters). However, bistable systems can, in principle, be constructed from diverse components and their interactions: transcriptional elements, enzymes, transport proteins, metabolic pathways, ligand binding, etc. A recent report compared bistable switches constructed with enzymatic feedback loops, feedback loops constructed from only transcriptional components and hybrid transcriptional/enzymatic switches [12]. It was found that enzyme-only circuits and hybrid transcriptional/enzymatic circuits showed bistable behavior for a wider parameter range than transcription-only circuits. This implies that hybrid bistable systems would likely be easier to tune and be more robust than systems built only from existing transcriptional components. In natural prokaryotic systems, the *lac* operon is arguably the most well-studied hybrid transcriptional/enzymatic bistable switch [21-23]. This system incorporates a lactose permease, enzymatic reactions, and transcriptional regulation. Once again, another well-studied hybrid bistable system is the *lux* operon in *Vibrio fischeri*[24], which uses an enzymatic reaction to convert a small molecule into an autoinducer to trigger transcriptional activation.

In this paper, we demonstrate a tunable family of hybrid bistable switches in *E. coli* using both transcriptional components and an enzymatic component (protease). We utilize standard transcriptional components to create a positive feedback loop. The behavior of the positive feedback loop, which normally does not show bistable behavior, is changed to a bistable network by adding a second feedback loop incorporating the Lon protease found in *Mesoplasma florum* (*mf*-Lon). To our knowledge, this work demonstrates the first use of *mf*-Lon to tune a nonlinear protein degradation circuit.

### Theory and design

#### Dynamic protein degradation and bistability

**Figure 1:**
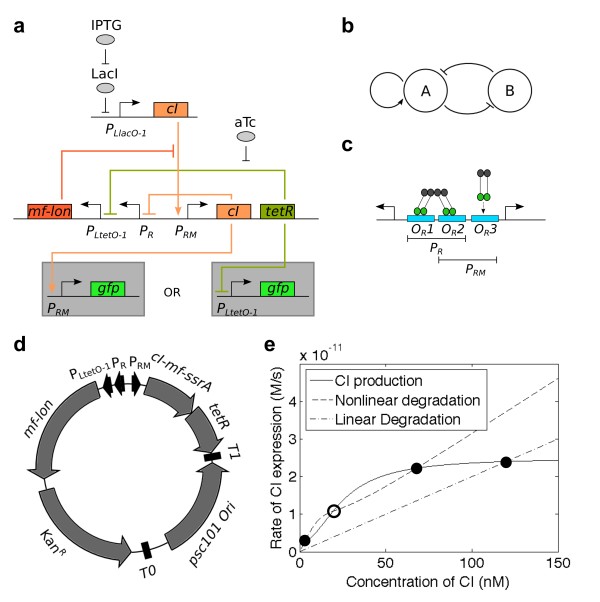
**Bistable switch utilizing dynamic protein degradation.** (**a**) The hybrid transcription/enzyme bistable switch consists of two promoters and three genes. (**b**) The basic topology can be shown as two linked positive feedback loops. The first positive feedback look consists of the autoactivation of *P*_*RM*_ by CI. The second positive feedback loop consists of TetR repression of *P*_*LtetO-1*_, and CI degradation by *mf*-Lon. The *P*_*R*_ and *P*_*LtetO-1*_ promoters are serially placed together, but effectively act as one promoter. (**c**) The *P*_*RM*_/*P*_*R*_ bidirectional double promoter consists of three CI dimer binding sites. CI dimers readily bind O_R_1 and O_R_2. O_R_3 is only bound when there are high concentrations of CI dimers. (**d**) The plasmid map shows that the bistable switch is built on a low copy number plasmid with pSC101 origin of replication and kanamycin marker. (**e**) The rate balance plot shows the difference between nonlinear degradation vs. standard linear degradation. The steady-state solutions to the concentrations of CI (and thus, system state) arise at the intersection of the CI production curve and either of the degradation curves. A linear degradation curve produces one steady-state solution. Nonlinear degradation creates three intersects with the CI production line to produce a bi-stable system. Solid circles indicate stable steady-states. Open circles represent unstable steady-states.

In this paper, we demonstrate the use of dynamic protein degradation to create a bistable switch. The switch (Figure1a) employs the Lon protease found in *Mesoplasma florum* (*mf*-Lon). This protease specifically degrades appropriately tagged proteins [25]. *Mf*-Lon works in much the same way as the more familiar *E. coli* ssrA degradation system, which utilizes the proteins ClpX and ClpP [26]. When translation stalls in *E. coli*, an ssrA tag is added to the C-terminus of the stalled polypeptide, which targets the polypeptide for degradation. Once the polypeptide is degraded, the ribosome is free to continue translation. The native *E. coli* degradation tag (*ec-*ssrA) is 13 amino acids long, while the *M. florum* degradation tag (*mf-*ssrA) is 32 amino acids. These two tags appear to be fully orthogonal and only the cognate proteases can recognize the proper tag [25]. *Mf*-Lon has been shown to function properly in *E. coli*, by degrading LacZ tagged with *mf*-ssrA [25]. We thus hypothesized that *mf*-Lon could be used in *E. coli* for dynamically targeting protein degradation by tagging specific proteins with *mf*-ssrA and dynamically expressing *mf*-Lon.

Other methods for engineering dynamic protein degradation exist but have disadvantages. In *E. coli*, knocking out the proteins ClpX and ClpP for dynamic expression compromises the fitness of knockout strains [27]. *B. subtilis* has a similar ssrA system as *E. coli*[28] and utilizes the same ssrA tags. Dynamic degradation of tagged proteins is possible in *B. subtilis* by using a mutated ssrA tag and dynamic expression of the *E. coli* gene, *ssbP*[29]. Proteins tagged with the mutated ssrA tag will only be degraded when *ssbP* is expressed. This method is plausible for organisms utilizing a native ssrA system similar to *E. coli* but missing the *ssbP* gene. Unfortunately, these conditions are difficult to meet, and may only be possible with *B. subtilis*. The use of a completely orthogonal tagging and degradation system, such as utilizing *mf*-Lon in *E. coli*, is thus superior to previously mentioned methods for engineering dynamic degradation of protein.

### Design of circuit

The switch is a hybrid of transcriptional components and enzymatic degradation (Figure 1a). It is comprised of two positive feedback loops: a positive auto-regulating loop and a loop with two antagonistic interactions (Figure 1b). Our positive auto-regulating loop is identical to one found in λ phage. The promoter P_*RM*_ expresses the λ repressor CI. CI is a transcriptional activator of P_*RM*_, thus producing positive feedback. The second positive feedback loop uses the tetracycline repressor (TetR) and *mf*-Lon. TetR is expressed bicistronically with CI in the P_*RM*_-CI loop. CI in the autoregulated loop is tagged with *mf*-ssrA (CI-*mf*-ssrA), allowing for fast degradation in the presence of *mf*-Lon. P_*LtetO-1*_ promoter [30] controls *mf*-Lon production. TetR is a repressor of P_*LtetO-1*_ and can stop *mf*-Lon expression, thus creating the co-antagonistic interaction; *mf*-Lon expression shuts off CI and TetR expression, while CI and TetR expression shuts off *mf-*Lon expression.

We defined the “on” versus “off” states of the switch in terms of the *P*_*RM*_-CI auto-regulation loop. When CI is produced, the switch is in the “on” state. When CI is degraded, the switch is “off.” We used superfolder GFP (sfGFP) [31] tagged with a fast degrading *ec*-ssrA tag controlled by P_*RM*_ (PRM-GFP)(see Table 1) to report the state of the switch. We also constructed another reporter plasmid, which expresses sfGFP with P_*LtetO-1*_(PLtetO-1-GFP). These two reporters were constructed on plasmids with p15A origins of replication and ampicillin markers. Both reporters were separately used to test the switch.

**Table 1:** List of Plasmids (accession numbers are listed in the supplemental section S.5)

**Name**	**Description**	**Marker**	**Origin**
PRM-GFP	GFP reporter expressed from P_*RM*_	Amp	p15A
PLtetO-1-GFP	GFP reporter expressed from P_*LtetO-1*_	Amp	p15A
PLlacO-1-CIwt	IPTG inducible CI-wt	Cm	ColE1
PLlacO-1-CILVA	IPTG inducible *CI-ec-*ssrA	Cm	ColE1
PLlacO-1-CImf	IPTG inducible CI-*mf*-ssrA	Cm	ColE1
RFP1	P_*LtetO-1*_ mf-lon (trunc) mCherry with RBS1	Amp	p15A
RFP2	P_*LtetO-1*_ mf-lon (trunc) mCherry with RBS2	Amp	p15A
RFP3	P_*LtetO-1*_ mf-lon (trunc) mCherry with RBS3	Amp	p15A
RFP4	P_*LtetO-1*_ mf-lon (trunc) mCherry with RBS4	Amp	p15A
RFP5	P_*LtetO-1*_ mf-lon (trunc) mCherry with RBS5	Amp	p15A
RFP6	P_*LtetO-1*_ mf-lon (trunc) mCherry with RBS6	Amp	p15A
RFP7	P_*LtetO-1*_ mf-lon (trunc) mCherry with RBS7	Amp	p15A
SW1	Full switch with 5-UTR1	Kan	pSC101
SW2	Full switch with 5-UTR2	Kan	pSC101
SW3	Full switch with 5-UTR3	Kan	pSC101
SW4	Full switch with 5-UTR4	Kan	pSC101
SW5	Full switch with 5-UTR5	Kan	pSC101
SW6	Full switch with 5-UTR6	Kan	pSC101
SW7	Full switch with 5-UTR7	Kan	pSC101
PRM-CIwt-TetR	CI-wt and TetR expressed from P_*RM*_	Kan	pSC101
PRM-CILVA-TetR	CI-*ec*-ssrA and TetR expressed from P_*RM*_	Kan	pSC101
PRM-CImf-TetR	CI-*mf*-ssrA and TetR expressed from P_*RM*_	Kan	pSC101
PLlacO-1-mfLon	IPTG inducible *mf*-Lon	Cm	ColE1

The use of TetR allows for induction using anhydrous tetracycline (aTc). When TetR is bound by aTc, TetR is rendered inactive and can no longer repress P_*LtetO-1*_ and results in *mf-*Lon expression. This causes CI degradation and forces the switch into the “off” state. CI expression can be forced with IPTG induction using a separate inducible plasmid (PLlacO-1-CImf). Inducing with IPTG forces the switch into the “on” state. Some previous systems incorporating CI in bistable networks invoked the SOS response to degrade CI to either flip states or reset the network as CI is quickly degraded through RecA mediated degradation [14,32]. Unfortunately, methods evoking the SOS response such as heat shock [14] or UV exposure [32] may be detrimental to cultures. Our system of utilizing *mf*-Lon allows for fast degradation of CI without evoking the SOS response.

CI also acts as a negative autoregulator at high concentrations [33] (Figure 1c). P_*RM*_ is half of a bidirectional promoter (Figure 1c) in λ phage. The complete promoter consists of three binding sites, O_R_1, O_R_2 and O_R_3. CI preferentially binds to O_R_1 and O_R_2, repressing the constitutive promoter, P_*R*_. In high concentrations, CI binds to O_R_3 and acts as a repressor to P_*RM*_. We used a mutated O_R_3 [34], in order to prevent autorepression.

The complete switch was constructed on a plasmid with a pSC101 origin and a kanamycin marker (Figure 1d). Both TetR and *mf*-Lon were tagged with a fast degradation *ec*-ssrA tag to allow for fast turnover rates for the proteins. P_*LtetO-1*_ has been placed serially next to P_*R*_ without separation by a terminator. It is unnecessary to use a terminator between these two promoters, because when the switch is in the “on” state, CI and TetR represses both P_*R*_ and P_*LtetO-1*_, respectively, and *mf*-Lon production is stopped. When the switch is in the “off” state both P_*R*_ and P_*LtetO-1*_ are not blocked and *mf-Lon* production is resumed. The serial placement of P_*LtetO-1*_ and P_*R*_ should not affect the basic principles governing the design of our switch.

### Conceptual comparison of hybrid bistable switch with transcriptional-only switch

A rate-balance plot [35] highlights the important differences between a fully transcriptional bistable switch (TS) (Supplemental Information S.1.5), and the hybrid transcriptional-enzymatic switch (TES) (Figure 1e). Both a TS and a TES use two linked positive feedback loops. The difference between the two topologies lies in the second positive feedback loop. In the TS, the second positive feedback loop uses two repressors. In the TES, the second positive feedback loop uses a repressor and a protease. The solid line on the rate balance plot (Figure 1e), represents the transcriptional activator production rate and the dashed curves represents the transcriptional activator degradation rate. A monostable system contains only one stable steady-state, represented by one intersection between the protein production and degradation curves. A bistable system requires two stable steady states (closed circles) and one unstable steady state (open circle), for a total of three intersections. In the “classic” TS, the addition of a second positive feedback loop adds nonlinearity to the CI production curve, resulting in a higher apparent Hill coefficient in the CI production curve (Additional file 1: S.1.5). Degradation rates for CI remain the same without the extra feedback loop and the degradation curve remains completely linear. In the TES, the addition of the second positive feedback loop, which contains the protease, changes the protein degradation from linear (dotted-dash line) to nonlinear (dashed line) while the CI production curve (solid line) remains unchanged (Figure 1e). The rate-balance plots show two different paradigms for creating bistable switches. One paradigm adds nonlinearity to activator production (TS), while the other adds nonlinearity to the activator degradation (TES).

Conceptually, TES topologies have the benefit of a larger bistable parameter space, allowing for increased robustness and easier parameter tuning [12]. Furthermore, enzymatic reactions have faster response times compared to transcriptional interactions. Enzymatic reactions directly deactivate proteins, by dephosphorylation, proteolysis, etc. In TS topologies, protein removal is also required to flip state after the deactivation of a promoter; this usually occurs through dilution by cell division or targeted protein degradation (e.g. *ssrA* tags). Increasing the rate of protein degradation in TS topologies allows for faster switching speeds, however, this further limits the already narrow parameter space of TS topologies.

Synthetic TS have been dominant in prokaryotic systems. This is likely due to the higher modularity and orthogonality that transcriptional parts have relative to enzymatic parts. Interconnections between transcriptional devices (a transcription factor and its cognate promoter) are simply created by adjacent placement in the DNA sequence of a regulated promoter and a transcription factor from two transcriptional devices. This allows for virtually any transcriptional device to serve as an input or output to any other transcriptional device. This contrasts with creating interconnections to inputs and outputs of enzymatic steps, where a common molecule is required and generally this need for a shared molecule greatly limits the number of parts an enzyme can be connected to. Moreover, many of the characterized enzymes are responsible for global housekeeping or are involved in metabolic pathways, limiting the applicability of any prokaryotic TES system relying on these enzymes. Proteases that are targeted to proteins with specific terminal sequences are not as constrained as other enzymes with respect to their modularity, as this class of proteases are not restricted to a single substrate, but can be used with any protein that will retain activity with the addition of the protease targeting signal.

## Results

### CI-mf-ssrA can activate P_RM_

First, it is necessary to check that the addition of the *mf*-ssrA tag onto the C-terminus of CI does not interfere with activation of P_*RM*_ by CI. The N-terminal domain of CI contains a DNA binding domain, and the C-terminal domain includes a dimerization domain [36]. CI first dimerizes and then binds onto O_R_1 and O_R_2 to activate P_*RM*_ and repress P_*R*_. Under normal physiological expression levels of CI the C-terminal domain is necessary for dimerization. Dimerization in turns helps with DNA binding. When using CI with a truncated C-terminal domain, higher concentrations of CI are necessary for dimerization to occur and thus higher CI concentration is necessary for DNA binding [37]. The addition of a degradation tag may interfere with proper protein interactions, and thus needs to be tested.

**Figure 2:**
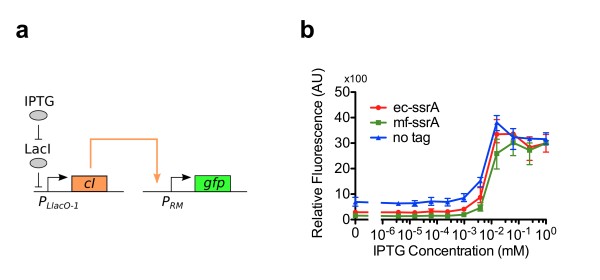
**CI turning on P**_***RM***_**.** (**a**) A two plasmid system was used to test the ability of *P*_*RM*_ activation by CI. This circuit consists of an IPTG inducible plasmid that expresses CI and a reporter plasmid expressing GFP by activation of P_*RM*_. Two different ssrA tags were added to the CI protein (*ec*-ssrA, *mf*-ssrA) to test the effect of ssrA tags on P_*RM*_ activation. (**b**) Fluorescence measurements were normalized to OD_600_ to account for cell density changes. Strains were cultured in various concentration of IPTG to test for P_*RM*_ activation. Error bars denote the measurement range (highest and lowest value). All data points were taken with three biological replicates.

CI tagged with an *E. coli* ssrA tag (CI-*ec*-ssrA) has been shown to not interfere with native activity [38]. CI-*ec*-ssrA also shows a shortened CI half-life [38,39]. Tagged CI has been widely used in various applications and retains activity [38,40]. We tested the ability of CI-*mf*-ssrA, CI-*ec*-ssrA, and untagged CI (CI-wt) to active P_*RM*_ (Figure 2a and b). In this test CI production was induced by IPTG, resulting in P_*RM*_ activation and GFP expression. All three variants of CI properly activated P_*RM*_. Across all IPTG concentrations, CI-wt had the highest GFP expression and CI-*mf*-ssrA generally had the lowest GFP expression.

As stated above, the activity of P_*RM*_ is not monotonic with CI concentration; above a threshold concentration of CI the activity of P_*RM*_ decreases with increasing CI. However, even for the P_*RM*_ promoter with mutated O_R_3 operator site, all three CI variants showed slight repression of P_*RM*_ at high levels of CI. CI-wt showed the highest degree of repression.

These results show the ability for CI-*mf*-ssrA to activate P_*RM*_, and thereby CI-*mf*-ssrA is usable for the proposed the TES. The results also show CI-*mf*-ssrA behavior slightly deviates from wild-type function; this will be discussed below.

### CI-mf-ssrA can be degraded by mf-Lon

**Figure 3:**
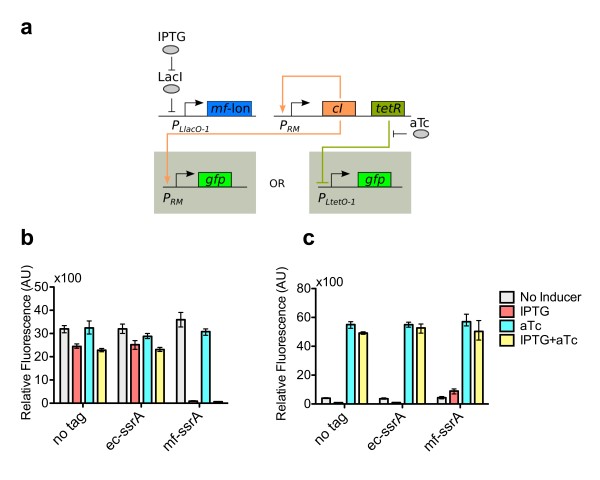
**Testing the*mf*-Lon protease.** (**a**) A three plasmid system was used to test CI degradation by *mf*-Lon. The circuit has a positive feedback loop consisting of P_*RM*_ expressing its activator, CI. Another plasmid expressed *mf*-Lon when induced with IPTG. Three different ssrA tag conditions were used for the activator (*ec*-ssrA, *mf*-ssrA, no tag). When *mf*-Lon is expressed a properly tagged CI should degrade, thus turning off the positive feedback loop. (**b**) The PRM-GFP reporter was used to measure the presence of CI. (**c**) ThePLtetO-1-GFP reporter was used to test the presence of TetR. All data points were measured using three biological replicates. Error bars indicate data range (highest and lowest value).

Orthogonality between the *M. florum* and *E. coli ssrA* systems has been demonstrated [25]. Given this, we investigated the ability of *mf*-Lon to degrade CI-*mf*-ssrA. We also needed to check for specificity between *mf*-Lon and its cognate tag (*mf*-ssrA). We tested the ability for *mf*-Lon to shut off the P_*RM*_-CI positive feedback loop (Figure 3a). We used the plasmids PRM-CIwt-TetR, PRM-CILVA-TetR, and PRM-CImf-TetR (Table 1) for this test; each plasmid contained a different CI/ssrA tag condition. This test circuit is almost the complete TES bistable network. The *mf*-Lon/TetR loop is open, so this test circuit does not form a bistable circuit. Instead, *mf*-Lon production was induced by IPTG, so *mf*-Lon production should be unaffected by both TetR and aTc concentrations. We utilized both GFP reporter plasmids with our test circuit. The PRM-GFP reporter plasmid more directly tested the P_*RM*_-CI loop state. The PLtetO-1-GFP plasmid reported on the TetR production level.

The P_*RM*_-CI positive feedback loop was functional using all three variants of CI (Figure 3b). All three CI variants, in the positive feedback loop configuration, showed strong levels of GFP expression (reporting with PRM-GFP). Expression of *mf*-Lon was only able to degrade CI-*mf*-ssrA and thus turn “off” the P_*RM*_-CI loop, resulting in greatly decreased GFP production. CI-wt and CI-*ec*-ssrA could not be degraded by *mf-*Lon and the GFP production did not change as drastically. This shows *mf-*Lon specificity to its cognate *ssrA* tag. In both the CI-wt and CI-*ec*-ssrA circuits, when IPTG addition was added, the P_*RM*_-CI loop remained “on,” however there was a measurable decrease in GFP expression. The decrease in GFP expression can probably be attributed to the increased metabolic burden from the expression of *mf*-Lon on a plasmid with a ColE1 origin of replication.

The PLtetO-1-GFP reporter tested TetR production level. TetR is biscistronically expressed with CI and the concentration of TetR should be approximately proportional to CI concentrations. When using the PLtetO-1GFP reporter, GFP expression increased only in the PRM-CImf-TetR test circuit (Figure 3c). This result again confirmed the specificity of *mf-*Lon to the *mf*-ssrA tag. GFP expression decreased with *mf*-Lon induction from test circuits expressing CI-wt and CI-*ec*-ssrA (Figure 3c). Once again, the decrease in GFP expression is likely due to increased metabolic load from *mf*-Lon induction.

The addition of aTc showed high GFP expression levels in all three experimental strains containing the three variants of CI (reporting with PLtetO-1-GFP). The addition of aTc inactivates TetR and *P*_*LtetO-1*_ becomes fully active regardless of *mf*-Lon or CI expression. Even when the P_RM_-CI positive feedback loop was shut “off” (resulting from CI-*mf*-ssrA degradation by *mf*-Lon), there was always basal TetR expression, which kept P_*LtetO-1*_ partially off. Only when aTc was added would P_*LtetO-1*_ become fully active. It is important to keep in mind that in these test circuits the TetR expression level is always high enough to have measurable effect on P_*LtetO-1*_ regardless of the P_*RM*_-CI state.

From these experiments we concluded that CI-*mf*-ssrA could indeed be degraded by *mf-*Lon. Just as important, *mf-*Lon specifically degrades proteins tagged with *mf-*ssrA and does not degrade proteins tagged with *ec*-ssrA.

### Creating a 5’-UTR library for mf-Lon

**Figure 4:**
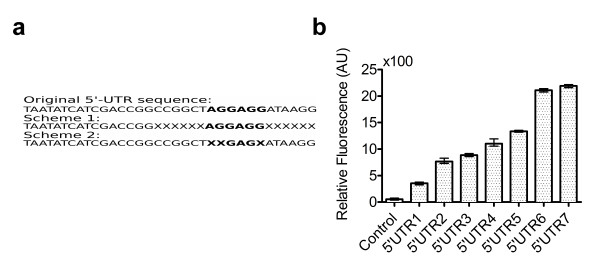
**Testing 5’-UTR strength.** (**a**) Two schemes were used to mutate and create the 5’-UTR library. Bold face type region indicates the Shine-Dalgarno consensus sequence. The top sequence shows the original sequence 5’UTR taken from the RBS calculator. The X’s in the schemes show the locations of random point mutations. (**b**) The 5’-UTR strength was estimated by measuring RFP fluorescence of a truncated *mf-Lon*::*mCherry* reporter using the respective 5’-UTRs numbered 1–7. The control strain does not express RFP. Data points were measured with three biological replicates. Error bars indicate data range (highest and lowest value).

We created a library of 5’-UTRs, which gave a range of *mf-*Lon expression levels. Initially the RBS calculator was used to create a 5’ untranslated region (5’-UTR) for the *mflon* gene [41]. Two different schemes were used to mutate the 5’-UTRs (Figure 4a). The first method randomized the 6 base pairs before and 6 base pairs after the Shine-Dalgarno (SD) region. The second method randomized three base pairs within the SD consensus region. To quantify the relative expression levels from the 5’-UTRs in the relevant context, these 5’-UTRs were added on a truncated *mflon* fused with *mCherry*[42]. This fusion protein was constitutively expressed using the P_*LtetO-1*_ promoter. Relative expression strength levels were assumed to be proportional to the measured RFP fluorescence of the fusion protein. A total of 192 colonies were screened for their level of RFP expression. Seven 5’-UTRs with different RFP expression levels (Figure 4b) spanning the complete RFP expression range of the library were selected and sequenced (Table 2). These seven 5’-UTRs were then incorporated into the switch construct. The 5’-UTRs were numbered 1–7 with 5’UTR1 being the weakest 5’-UTR and 5’UTR7 being the strongest 5’-UTR. The switches incorporating the respective 5’-UTR were named in the same fashion (e.g. SW1 refers to a switch which incorporates 5’UTR1).

**Table 2:** 5’UTR sequences

**Name**	**Sequence**
Original	TAATATCATCGACCGGCCGGCT**AGGAGG**ATAAGG
5’UTR1	TAATATCATCGACCGGCCGGCT**CCGAGC**ATAAGG
5’UTR2	TAATATCATCGACCGGCCGGCT**CAGAGC**ATAAGG
5’UTR3	TAATATCATCGACCGGCCGGCT**AAGATA**AGG
5’UTR4	TAATATCATCGACCGGCCGGCT**GAGAGA**ATAAGG
5’UTR5	TAATATCATCGACCGGCCGGCT**ACGAGG**ATAAGG
5’UTR6	TAATATCATCGACCGGCCGGCT**AGGAGG**ATAAGG
5’UTR7	TAATATCATCGACCGGAGAAGA**AGGAGG**TGCTGGT

### Tuning mf-Lon production with RBS strength yields bistable switches

**Figure 5:**
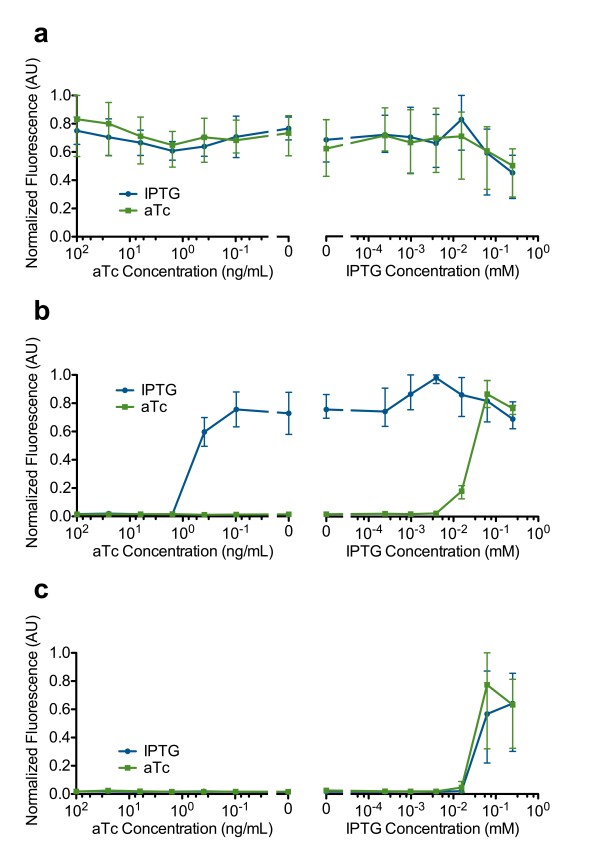
**Observing hysteresis to test for bistability.** Hysteresis was measured using the PRM-GFP reporter. Blue curves represent samples cultured overnight in IPTG. Green curves represent samples culture overnight in aTc. (**a**) SW3 was “always on.” (**b**) SW4 had bistable behavior. (**c**) SW7 was “always off.” Each data point was measured with three biological replicates. Error bars represent the data range (highest and lowest value).

The presence or absence of bistable behavior was determined by measuring hysteresis. SW1, SW2 and SW3 were assayed along using the PRM-GFP reporter plasmid and showed “always on” behavior, indicating *mf*-Lon expression was too low to turn off the P_*RM*_-CI loop. Even with full TetR inactivation using saturating levels of aTc, the P_*RM*_-CI loop remained “on.” No hysteresis was measured in SW1, SW2 and SW3. The behavior of SW3 (Figure 5a) is representative of SW1, SW2 and SW3.SW4, SW,5 and SW6 with PRM-GFP displayed bistable behavior. The behavior of SW4 (Figure 5b) is representative of SW4, SW,5 and SW6. For SW4, SW,5 and SW6 we observed a bistable region at low levels of inducers (0–0.4 ng/mL aTc and 0–0.016 mM IPTG). At high levels of inducer we observed monostability where the two curves converged. Table 3 summarizes the behavior of SW1 through SW7.

**Table 3:** **Switch behavior (in the absence of inducers**)

**Name**	**Behavior**
SW1	Monostable “on”
SW2	Monostable “on”
SW3	Monostable “on”
SW4	Bistable
SW5	Bistable
SW6	Bistable
SW7	Monostable “off”

SW7 was assayed using with the PRM-GFP reporter plasmid and showed monostable behavior; the switch was always off unless high levels of IPTG were used to induce high levels of CI production (Figure 5c).

**Figure 6:**
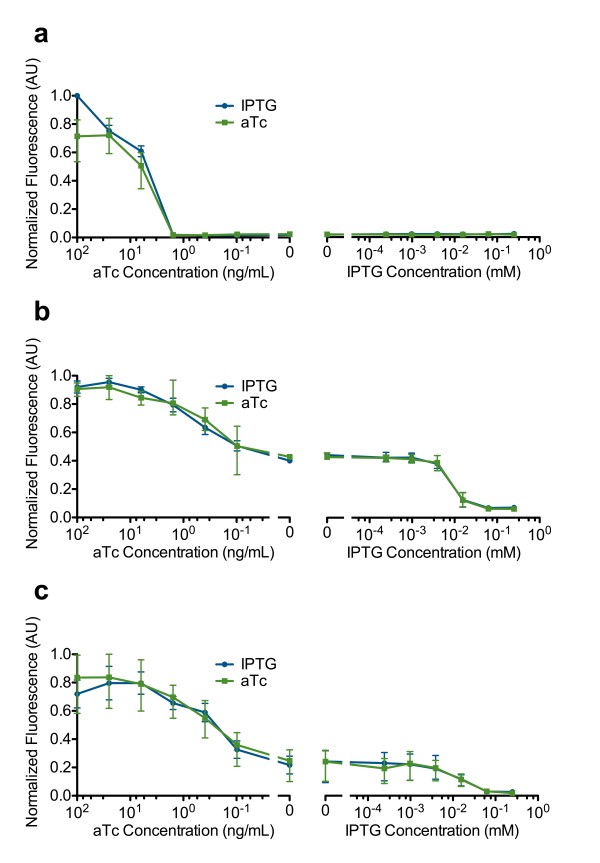
**P**_***LtetO-1***_**activity in the bistable switch.** P_*LtetO-1*_ activity was measured using the PLtetO-1-GFP reporter. Blue curves represent samples cultured overnight in IPTG. No hysteresis was measured in (**a**) SW3, (**b**) SW4, or (**c**) SW7. Each data point was measured with three biological replicates. Error bars represent the data range (highest and lowest value).

Hysteresis was not observed for any of the switches when using the PLtetO-1-GFP reporter (Figure 6). This however does not indicate the lack of bistable behavior in SW4, SW5 and SW6. The lack of hysteresis observed using the PLtetO-1-GFP reporter is discussed later.

### Both states of a tuned switch are stable over 40 doublings

**Figure 7:**
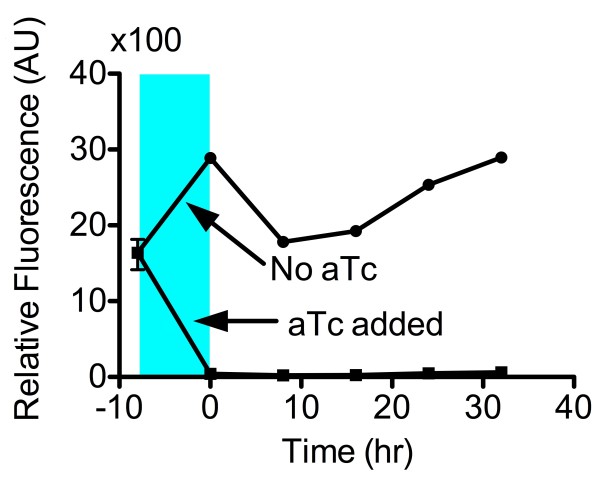
**Testing for long term stability.** SW4 was tested to show its ability to hold both the “on” and “off” states for over 30hr. SW4 initially was in the “on” state. SW4 was then split into two subcultures where one subculture was grown with aTc (blue region), and the other subculture was grown without inducer. At time t=0, inducer was removed, and cultures were grown and repeatedly subcultured for approximately 40 cell divisions.

A long-term stability test was performed on SW4 to check for the stability of both states. The strains were able to hold their states throughout the 32 hour period (~40 cell divisions) (Figure 7).

### Transient behavior of switches

**Figure 8:**
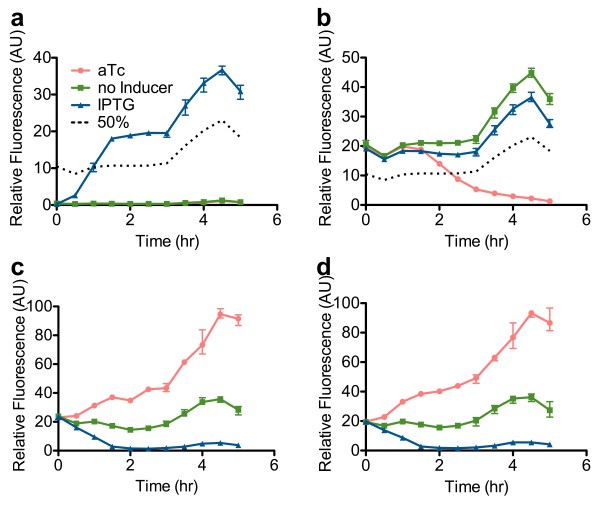
**Transient switching behavior.** Transient behavior was measured for (**a**) SW4 starting in the “on” state, and (**b**) starting in the “off” state using the PRM-GFP reporter. Transient behavior was measured for (**c**) starting in the “on” state, and (**d**) starting in the “off” state using the PLtetO-1-GFP reporter. At time t = 0 inducers are added into the culture to either reinforce the state, alter the state, or allow the state to persist (no inducer added). Each data point was measured using three biological replicates. Error bars indicate the data range (highest and lowest value). The 50% line measures the 50% point between maximum and minimum GFP expression. Because the baseline “on” and “off” GFP expression changes over time as OD increases, the 50% level also changes with OD. The 50% line is used to measure the activation and deactivation time. See methods section for more details on measuring the 50% line.

We tested the transient behavior of SW4, SW5 and SW6. Both GFP reporters were used in this set of experiments. We tested for activation time and deactivation time using the PRM-GFP reporter. Activation time (ACT½) was defined as the time necessary for GFP expression to go from the initial low expression to 50% expression. Deactivation time (DCT½) was defined as the time necessary for GFP expression to decrease to 50% expression. Each strain was initially forced into either the “on” or “off” state. Next, inducers were removed and strains were allowed to reach steady-state. The appropriate inducer was then added to change states. We also measured the behavior when no inducer was added in order to observe transient state persistence. Lastly, we also measured transient behavior when inducer was added to reinforce the state (e.g. adding aTc to a switch already in the “off” state). Only results for SW4 are shown (Figure 8). Results for SW5 and SW6 are shown in Additional file 1: S.2.

When no inducer was added, each strain was able to hold its previous state. When inducer was added to reinforce the state, each strain kept its previous state. Both the “reinforcement” and “no inducer” curves were similar. When inducer was added to change the state, we quantified the ACT½ and DCT½ (Table 4). ACT½ was similar for SW4, SW5 and SW6 with all three switches reaching 50% activation in approximately 1 hour. DCT½ was as expected, with SW4 having the longest DCT½ (139 min) and SW6 having the shortest (83 min).

**Table 4:** Activation deactivation times for 3 biological replicates

**Name**	**Average ACT½**	**ACT½ Range**	**Average DCT½**	**DCT½ Range**
SW4	59 min	55–64 min	139 min	137–140 min
SW5	53 min	48–57 min	90 min	82–98 min
SW6	65 min	61–67 min	83 min	81–85 min

Activation was dependent on CI accumulation rate. To first order, the CI accumulation rate is dependent on CI production rate. All strains had the same inducible CI plasmid, and identical P_*RM*_-CI positive feedback loops. As a result, all three switches should have the same CI production rates. As observed, ACT½ for all three strains did not differ by much (as compared to DCT½). Protein degradation rate is dependent on protease concentration, giving the expected results for deactivation time. Once again when we reported SW4, SW5 and SW6 with PLtetO-1-GFP (Figure 8c and d), no hysteresis was observed. The curves in the “on” state or “off” state are identical.

Given the fast deactivation of SW6, the dynamic measurements of SW6 were repeated using flow cytometry (Supplemental Information S.3.1) to ensure that the “on” state and “off” state were each composed of a unimodal distribution of cells in that state. The flow cytometry data confirms that each state contains a unimodal distribution of cells.

## Discussion

We present a new bistable topology in *E. coli*. Our system specifically uses an ssrA degradation system orthogonal to the native *E. coli* system. The usage of the *mf-*Lon protease and *mf-*ssrA tag in a useful circuit had not previously been explored. The usage of nonlinear protein degradation in a bistable switch has mainly been a theoretical discussion and has not been synthetically employed. In this paper we experimentally tested the effect of using nonlinear protein degradation in a synthetic prokaryotic system.

### CI-mf-ssrA degradation

We found that CI-wt activates P_*RM*_ the most strongly as compared to CI with ssrA tags (Figure 2b). It is known that the CI dimer is extremely stable and has a long half-life [17]; even with low expression rates CI-wt can quickly accumulate and activate P_*RM*_. The *mf-*ssrA tag should be completely orthogonal to the *ec*-ssrA tag [25]. In *E. coli*, the half-life of proteins tagged with *mf*-ssrA should be unaffected since they will not be targeted for degradation. We report that CI-*mf*-ssrA has a different behavior than CI-wt. CI-*mf*-ssrA requires a higher expression rate to activate P_*RM*_ compared to CI-wt. It requires a higher expression rate than even CI-*ec*-ssrA. This implies that CI-*mf*-ssrA is intrinsically unstable and degrades quickly, even when it is not targeted for degradation. Most likely the addition of 27 aa ssrA tag interferes with the C-terminal domain of the lambda repressor, as opposed to the 13 aa *ec*-ssrA tag. The *mf*-ssrA tag disrupts the C-terminal domain dimerization of CI, requiring a higher concentration of CI for dimerization. Furthermore, CI dimers are cooperatively stabilized. Unless there are high expression levels of CI, few CI dimers are formed and the unstable CI monomers are quickly degraded [17]. Since *mf-*ssrA can interfere with protein functionality, this implies that the number of proteins that can be used with the *mf-*ssrA degradation system may be limited.

### Controlling switching speeds by dynamically expressing a protease

Enzyme substrate reaction rates are dependent on both the concentration of the enzyme and the substrate. The maximum reaction rate, V_max_, is directly proportional to enzyme concentrations. If switching speeds need to be modified, adjusting protease expression levels provides an easy way to tune the switching speed. In our reported TES, changing protease expression levels only changed the deactivation speed. The activation speed is most likely controlled by changing the CI expression rate on the PLlacO-1-CImf plasmid (this was not explicitly tested).

It should be noted that on TS, changing the expression levels of transcriptional activators and repressors does not change the activation and deactivation speeds significantly, because protein degradation is the limiting factor and is unchanged by protein expression rates. The addition of degradation tags in a TS may not be an option due to parameter constraints. In our TES, protein degradation is not a limiting factor, because proteins are either tagged with a fast degradation tag or dynamically degraded by a protease, which allows for flexibility in changing activation and deactivation speeds.

The TES topology presented can be rendered modular by using two input promoters. One promoter would express CI and the other would express *mf*-Lon. In this scheme DCT½ would depend on both the expression rate of *mf-*Lon from the linking circuit as well as the expression rate on the switch itself. Depending on relative rates within the switch and on the input, the faster rate would dominate the other. The switching “on” rate would be dominated by CI production rate on the input circuit.

### The lack of hysteresis from P_LtetO-1_

Both the hysteresis data and transient behavior data confirmed the lack of hysteresis from P_*LtetO-1*_. When using the PLtetO-1-GFP reporter, flow cytometry experiments showed that in the absence of inducers, cells in either the “on” or “off” state had identical GFP expression levels (Additional file 1: S.3.3). With the addition of aTc we observed high levels of GFP expression in a tightly distributed unimodal population (Additional file 1: S.3.2). When IPTG was added, there appeared to be a bimodal population of cells; one population did not express GFP, the other population expressed low levels of GFP. The bimodal population of cells implied that TetR did not completely repress P_LtetO-1_. However, TetR production was clearly high enough to prevent a fully active P_*LtetO-1*_ promoter. We saw that even when the switch was “off,” there was enough TetR expression to keep P_*LtetO-1*_ partially off and only with the addition of aTc would P_*LtetO-1*_ be fully active. It is unknown why the addition of IPTG was unable to completely shut off P_*LtetO-1*_.

### Implications regarding the lack of hysteresis from P_LtetO-1_

One last observation is that the observed lack of hysteresis in *mf*-Lon concentration may make it unnecessary to add a second positive feedback loop to create a bistable network. Instead, bistable behavior may be possible using only a single positive feedback loop along with constitutive expression of *mf*-Lon. The simplified circuit would consist of a P_*RM*_-CI positive feedback back loop, with CI tagged with *mf*-ssrA. The expression level of *mf*-Lon would determine the degradation rate of CI-*mf-*ssrA (Supplemental Information S.4.3). Using the literature values of the reported Michaelis constant for *mf*-Lon (*K*_m_) [25] and the literature value for the dissociation constant of CI-wt to P_*RM*_ binding (*K*_dCI_) [33], bistable behavior cannot be obtained with a single positive feedback loop. Using modified *K*_m_ where *K*_*dCI*_>*K*_*m*_, bistability is analytically observed using a rate balance plot (Additional file 1: S.4.4).

The literature value for *K*_*m*_ may be inaccurate, since *K*_m_ was measured in vitro. The *in vivo* value may actually be lower value than the measured in vitro *K*_*m*_ value. The native function of *mf*-Lon is to rescue stalled transcription events. With a high *K*_*m*_, stalled events cannot be rescued efficiently. Natively, *mf*-Lon is a housekeeping gene, and expression of the protease is probably low. The *in vivo* functioning of *mf*-Lon is most likely fast and efficient, even at low protease concentrations. In order to create the bistable rate-balance plot, *K*_*m*_ needs to be decreased by three orders of magnitude, and this shift in value is also highly doubtful. The lack of hysteresis for *P*_*LtetO-1*_ and its implications require more tests to be fully understood.

### Comparison of a degradative a TES vs the tradition TS

The principal benefit of the protein degradative interaction in the TES is the tunability of switching speeds due to active removal of proteins through a protease. In the TS, removal of proteins is through dilution by cell division. The original toggle [14] switch required 3–4 hrs for to begin switching, and over 5 hours for the switch to completely change states. Switching states in the linked double positive feedback loop reported by Chang *et al.*[10] required the dilution of both proteins and the small molecule IPTG. In this case switching required a 1 million-fold cell dilution for proper switching. With active protein removal through a protease we were able to complete switching in approximately 3 cell divisions (~2.5 hr). In theory, switching could be accomplished within one cell division. This would require the simultaneous tuning of the RBS for all three proteins (CI, TetR and *mf*-Lon) to optimize deactivation time.

Another benefit of our TES compared to a TS is the increased robustness of the TES. It has been previously shown that these types of hybrid circuits are more robust than transcription-only circuits [12]. Adding a protease introduces nonlinear degradation and thereby adds nonlinearity into the system. In classic TS systems, some nonlinearity can be added by increasing the apparent cooperativity in the main positive feedback loop. Additionally, it is difficult to add ec-ssrA tags in the TS, since the parameter space is already very small. Shortening the protein lifetime also decreases the robustness of the circuit. In the TES, the parameter space was broad enough to allow for the addition of ec-ssrA tags. It can be argued that increased bistable parameter space contributes to the tunability of switching time.

## Conclusions

We have demonstrated a hybrid bistable switch using both transcriptional components and an enzyme. Bistable behavior is achieved through nonlinear degradation of the transcriptional activator. A dynamically expressed ssrA protease orthogonal to the *E. coli* system was used in order to achieve nonlinear protein degradation. Use of a protease allows for an easy way to tune switching speed in bistable circuits. This is a new method of creating synthetic bistable networks, which have been theoretically proposed, but have not been tried experimentally.

## Materials and Methods

### Bacterial strains, media, and growth conditions

Cloning steps and experiments were performed with *E. coli* strain DH10B. Overnight cultures were performed in Luria-Bertani (LB) medium at 37°C. All experiments were performed in EZ-rich media with 1% glucose, purchased from Teknova and were grown at 37°C. Antibiotics were used with the following concentrations: kanamycin at 40 μg/mL, chloramphenicol at 20 μg/mL, ampicillin at 100 μg/mL, carbenicillin at 100 μg/mL. Experiments used carbenicillin in lieu of ampicillin. Cloning steps used ampicillin instead of carbenicillin. Restriction enzymes were purchased from Fermentas Inc. Polymerases were purchased from Finnzymes and Stratagene. Media, antibiotics and enzymes were used according to manufacturer recommendations.

### Plasmid construction

Table 1 lists all the plasmids used in this study. All cloning plasmids in this study have the BglII/BamHI BglBrick sites [43]. Ribosome binding sites (RBS) were added using ‘Round-the-horn site directed mutagenesis (RTH) and its respective primers [44]. RBS were designed using the RBS calculator. IPTG inducible plasmids with Cm marker were constructed by inserting the respective genes into BglBrick sites using the XhoI and BglII restriction sites. *Mf*-ssrA and *ec*-ssrA tags (AANDENYALVA) [45] were added using RTH and its respective primers. CI-*ec*-ssrA, sfGFP, TetR and *mf*-Lon were destabilized with the addition of a *ec-ssrA* tag.

*Mf-lon* was obtained from Addgene (Plasmid 21867: pBAD33-mf-lon) [25]. The sequence was amplified with PCR adding BglBrick sites then added into a high copy cloning plasmid with pUC19 origin of replication and ampicillin marker. Superfolder GFP (sfGFP) is the same as described by Pedelacq *et al.*[31]. The RFP, *mCherry*, is described by Shaner *et al.*[42].

### Florescence measurements for endpoint measurements

Endpoint measurements started with a 5 mL LB overnight culturing with 200 rpm of continuous shaking. After overnight culturing each sample was subcultured with a 100 fold dilution into 5 mL of EZ-rich media and regrown to an OD_600_ of 0.4. The cultures were then subcultured into EZ-rich media to an OD of 0.05 with added inducers. Florescence measurements were measured by transferring 150μL of culture into a 96 well microplate to measure OD_600_ and fluorescence on a Molecular Devices Spectramax M2. GFP measurements used 485 nm for excitation and 538 nm for emission. RFP measurements used 584 nm for excitation and 612 nm for emission. All fluorescence measurements were normalized to OD_600_. All reported measurements were repeated in triplicate by using three different colonies.

### Hysteresis measurements

A single colony was picked out a plate transformed with the relevant plasmids and cultured overnight in both 5 mL LB with IPTG (.0625 mM) and 5 mL LB with saturating aTc (100 ng/mL) to force them into the high and low state respectively. Generally 1 mM of IPTG was used for saturating conditions. We found that reduced levels of IPTG were sufficient to induce high levels of CI and push switches into the “on” state. The reduced levels of IPTG also allowed for easier removal of IPTG. The overnight cultures were washed three times with EZ-rich media and split into multiple subcultures on a 96 deep well plate. Each subculture was grown with a total of 1 mL of media and the plate was shaken at 250 rpm. More specifically each overnight sample was subcultured into 7 different IPTG conditions (ranging from 0 mM to .25 mM) and 7 different aTc conditions (ranging from 0 ng/mL to 100 ng/mL). After cell growth reached mid-log (~8 hrs), each sample on the 96 well plate was recultured on a new 96 well plate with fresh media using exactly the same inducer concentrations as the previous 96 well plate. This step ensured complete removal of the initial inducer. After the samples reached mid-log again, GFP output was measured. For each switch, GFP output was measured for both PLtetO-1-GFP and PRM-GFP reporters. Each data point was replicated three times using three individual bacterial colonies.

### Long term stability experiment

The long term stability test was performed only on SW4 with the PRM-GFP reporter. SW4 was grown in 5 mL LB overnight with saturating IPTG (.0625 mM). The overnight culture was washed three times and recultured in fresh EZ-medium using a 1000 fold dilution. After an OD_600_ of 0.5 was reached the culture was then split into two subcultures. Both were cultured with 1000 fold dilutions. One culture had 100 ng/mL of aTc added in order to switch in to the “off” state. The other culture was grown without aTc. After 8 hr of growth, both samples were washed and subcultured in fresh media without any inducer, again with a 1000 fold dilution. Both samples were grown an additional 32 hr, washing and subculturing every 8 hr, again using 1000 fold dilutions. GFP measurements are taken every eight hours before each subculturing event. The doubling time was approximately 45 minutes, giving approximately 10 doubling every 8 hr. The total experiment had 40 doublings throughout the stability measurement. Each data point was repeated three times using three separate colonies.

### Transient switching measurements

Transient measurements started with overnight cultures in 5 mL LB. One colony was picked from an agar plate to inoculate two 5 mL LB culture tubes. One culture tube had 100 ng/mL of aTc, and the other tube had 0.0625 mM of IPTG. After overnight growth (16 hr), the overnight cultures were washed three times in EZ-rich media and subcultured with 1000 fold dilution in EZ-rich media and regrown to an OD_600_ of approximately 0.8. Each culture was split into three separate subcultures each with a different inducer (0.25 mM IPTG, 100 ng/mL aTc, and no inducer) and diluted down to an OD_600_ of 0.05. The three different inducer conditions represent three transient conditions (change, reinforce, and persist). “Change” is when an inducer is added to alter the state. “Reinforce” is when the inducer used is the same as the overnight inducer. “Persist” is when no inducer is added and switches are allowed to keep their prior state. Fluorescence measurements were taken every 30 minutes. Measurements were all performed in triplicate using three separate colonies. GFP expression was reported using both reporters.

To measure 50% activation (ACT½), we measured the time it took for a strain, initially in the “off” state, to reach 50% of maximum GFP expression. Because the baseline “on” and “off” GFP expression changes over time as OD increases, the 50% level also changes with OD. The 50% level is found by taking the average value between the “on persist state” and “off persist state” for each time point. The 50% deactivation (DCT½) level is found in a similar fashion as the 50% activation calculation. ACT½ and DCT½ were only measured using PRM-GFP. ACT½ and DCT½ were measured three times using three separate colonies.

### Flow cytometry

Cultures to be measured were diluted 1:2000 in phosphate buffered saline (from Teknova) with 50 μg/mL of streptomycin. 150 mL of the diluted sample was placed in a 96 well microplate, and then measured on a Milipore guava easyCyte HT. For each sample approximately 2000 events were counted.

## Competing interests

The authors declare that they have no competing interests.

## Author contributions

DH and WH conceived of the experiments. DH performed the experiments. DH, WH and MM wrote the manuscript. All authors read and approved the final manuscript.

## Supplementary Material

Additional file 1 **S.1 Comparison of TS and TES circuits [**[46-51]**].** Click here for file
