# Supplementary material for: A genetic bistable switch utilizing nonlinear protein degradation
Source: J Biol Eng. 2012 Jul 9;6:9. doi: 10.1186/1754-1611-6-9 (PMC3439342; doi:10.1186/1754-1611-6-9)
Supplement: Additional file 1 — S.1 Comparison of TS and TES circuits [[46-51]]. [file 1754-1611-6-9-S1.pdf]

## Supplemental Information

## S.1 Comparison of TS and TES circuits

### S.1.1 Equations for bistable TES

This set of equations describes the bistable TES in figure 1 of the main paper. The parameters values used to produce the rate-balance plot in figure 1e is shown in S.1.3

$$\frac{d}{dt}m_{cI} = V_{P_{RM}}NC\left[\frac{1}{1+\left(\frac{K_{CI}}{p_{CI}}\right)^{nr}} + \ell_{P_{RM}}\right] - \gamma_m m_{cI} \tag{1}$$

$$\frac{d}{dt}p_{CI} = \epsilon_{CI}m_{cI} - \gamma_{CI}p_{CI} - \frac{p_L k_{cat}}{1+\frac{K_M}{p_{CI}}} \tag{2}$$

$$\frac{d}{dt}m_t = V_{P_{RM}}NC\left[\frac{1}{1+\left(\frac{K_{CI}}{p_{CI}}\right)^{nr}} + \ell_{P_{RM}}\right] - \gamma_m m_t \tag{3}$$

$$\frac{d}{dt}p_T = \epsilon_T m_t - \gamma_T p_T \tag{4}$$

$$\frac{d}{dt}m_l = V_{P_{LtetO-1}}NC\left[\frac{1}{1+\left(\frac{p_T}{K_T}\right)^{nt}} + \ell_{P_T}\right] - \gamma_m m_l \tag{5}$$

$$\frac{d}{dt}p_L = \epsilon_L m_l - \gamma_L p_L \tag{6}$$

## S.1.2 Equations for bistable TS

This set of equations describes the bistable TS show in S.1.4 and rate-balance plot S.1.5. The parameters values used to produce the rate balance plot in S.1.5 are shown in S.1.2.

$$\frac{d}{dt}m_{cI} = V_{P_{RM}}NC\left[\frac{1}{\left\{1+\left(\frac{K_{CI}}{p_{CI}}\right)^{n_r}\right\}\left\{1+\left(\frac{p_{CI434}}{K_{434}}\right)^{n_{434}}\right\}} + \ell_{P_{RM}}\right] - \gamma_m m_{cI} \qquad (1)$$

$$\frac{d}{dt}p_{CI} = \epsilon_{CI}m_{cI} - \gamma_{CI}p_{CI} \qquad (2)$$

$$\frac{d}{dt}m_t = V_{P_{RM}}NC\left[\frac{1}{\left\{1+\left(\frac{K_{CI}}{p_{CI}}\right)^{n_r}\right\}\left\{1+\left(\frac{p_{CI434}}{K_{434}}\right)^{n_{434}}\right\}} + \ell_{P_{RM}}\right] - \gamma_m m_t \qquad (3)$$

$$\frac{d}{dt}p_T = \epsilon_T m_t - \gamma_T p_T \qquad (4)$$

$$\frac{d}{dt}m_{CI434} = V_{P_{LtetO-1}}NC\left[\frac{1}{1+\left(\frac{p_T}{K_T}\right)^{n_t}} + \ell_{P_T}\right] - \gamma_m m_{CI434} \qquad (5)$$

$$\frac{d}{dt}p_{CI434} = \epsilon_{CI434}m_{cI434} - \gamma_{CI434}p_{CI434} \qquad (6)$$

## S.1.3 List of parameters

| Parameters and Variables | Description | Units | Value for TES | Value for TS |
|---|---|---|---|---|
| $m_{cI}$ | mRNA concentration for *cI* | $M$ | | |
| $m_t$ | mRNA concentration for *tetR* | $M$ | | |
| $m_l$ | mRNA concentration for *mf-lon* | $M$ | | |
| $p_{CI}$ | Protein concentration for CI | $M$ | | |
| $p_T$ | Protein concentration for TetR | $M$ | | |
| $p_L$ | Protein concentration for *mf*-Lon | $M$ | | |
| $V_{P_{RM}}$ | Velocity for mRNA production for $P_{RM}$ | $M \cdot s^{-1}$ | .3 [46] | .3 [46] |
| $V_{P_{LtetO-1}}$ | Velocity for mRNA production for $P_{LtetO\text{-}1}$ | $M \cdot s^{-1}$ | .3 [30] | .3 [30] |
| $\ell_{P_{RM}}$ | mRNA leakage of $P_{RM}$ promoter | $M \cdot s^{-1}$ | 1/10 [47] | 1/10 [47] |
| $\ell_{P_T}$ | mRNA leakage of $P_{LtetO\text{-}1}$ promoter | $M \cdot s^{-1}$ | 1/5050 [30] | 1/5050 [30] |
| $K_{CI}$ | Disassociation constant of CI to $P_{RM}$ | $M$ | $2.5\times10^{-8}$ [33] | $2.5\times10^{-8}$ [33] |
| $K_{CI434}$ | Disassociation constant of CI434 to $P_{RM}$ | $M$ | | $2.5\times10^{-8}$ [32] |
| $K_T$ | Disassociation constant of TetR to $P_{LtetO\text{-}1}$ | $M$ | $1.79\times10^{-10}$ [48] | $1.79\times10^{-10}$ [48] |
| $K_m$ | Michaelis constant for *mf*-Lon | $M$ | $3.7\times10^{-6}$ [25] | $3.7\times10^{-6}$ [25] |
| $k_{cat}$ | Catalytic rate of *mf*-Lon | $s^{-1}$ | .071 [25] | .071 [25] |
| $\gamma_m$ | Degradation rate of mRNA | $s^{-1}$ | $2.38\times10^{-3}$ [49] | $2.38\times10^{-3}$ [49] |
| $\gamma_{CI}$ | Degradation rate of CI | $s^{-1}$ | $2\times10^{-4}$ | $2\times10^{-4}$ |
| $\gamma_{CI434}$ | Degradation rate of CI434 | $s^{-1}$ | | $2\times10^{-4}$ |
| $\gamma_T$ | Degradation rate of TetR | $s^{-1}$ | $7\times10^{-4}$ [39] | $2\times10^{-4}$ |
| $\gamma_L$ | Degradation rate of *mf*-Lon | $s^{-1}$ | $7\times10^{-4}$ [39] | $2\times10^{-4}$ |
| $\epsilon_{CI}$ | Translation rate for CI | $s^{-1}$ | $4.5\times10^{-5}$ | $3\times10^{-5}$ |
| $\epsilon_{CI434}$ | Translation rate for CI | $s^{-1}$ | | $5\times10^{-5}$ |
| $\epsilon_T$ | Translation rate for TetR | $s^{-1}$ | $3\times10^{-8}$ | $6\times10^{-7}$ |
| $\epsilon_L$ | Translation rate for *mf*-Lon | $s^{-1}$ | $3.5\times10^{-5}$ | |
| $n_r$ | Hill coefficient for $P_{RM}$ | | 2[50] | 2[50] |
| $n_{434}$ | Hill coefficient for $P_{RM}$ | | 1[50] | 1[50] |
| $n_t$ | Hill coefficient for $P_{LtetO\text{-}1}$ | | 2[50] | 2[50] |
| $N$ | Plasmid copy number for psc101 | | 3 | 3 |
| $C$ | Concentration of a single protein/mRNA in a typical bacterium | $M$ | $1.5\times10^{-9}$[51] | $1.5\times10^{-9}$[51] |

It should be noted the degradation rate of all proteins in TS is the doubling time of the cell, indicating the absence of *ssrA* degradation tags.

### S.1.4 Schematic for TS

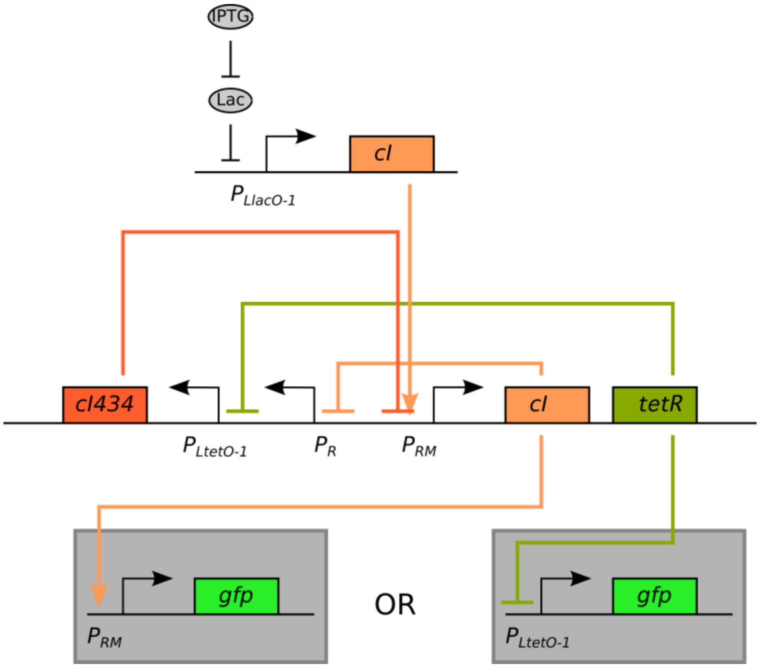


Figure S.1 – Bistable TS

The full transcriptional bistable switch consists of two promoters and three genes, and is based off of our bistable TES from figure 1 of the main paper. The repressor CI434 is used in place of *mf*-Lon. In order for CI434 to repress $P_{RM}$, $O_R3$ needs to be changed to a CI-434 binding site [32]. Just as in TES, the main positive feedback loop is the $P_{RM}$-CI loop. The second loop consists of $P_{LtetO-1}$ making CI434, repressing $P_{RM}$.

### S.1.5 Rate balance plot for TS plot

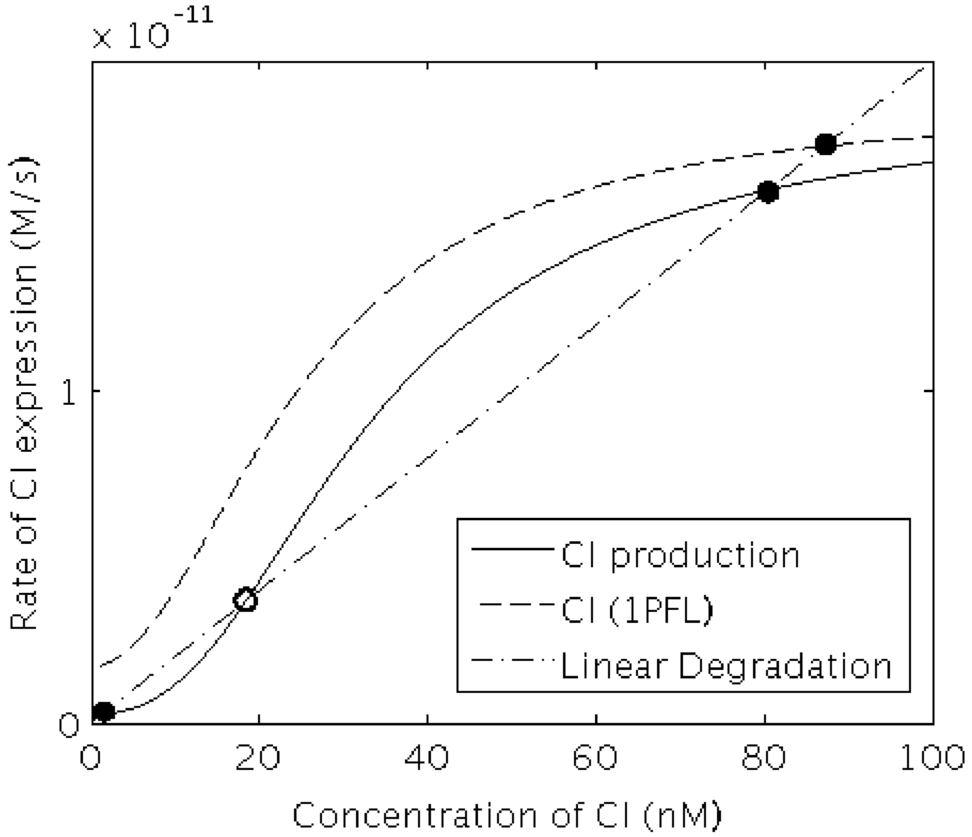


Figure S.2 – Rate-balance plot of a bistable TS

Just like for the bistable TES, having only the $P_{RM}$-CI positive feedback loop, bistabile behavior is not possible. Bistable behavior is conferred once the second positive feedback loop. In the degradative TES, the linear degradation of CI is changed into a nonlinear degradation to create a bistable condition (figure 1e). With the TS, bistability was created by adding more nonlinearity into the CI production rate (figure S.2, dashed line).

## S.2 Kinetic Data for SW5 and SW6 reporter

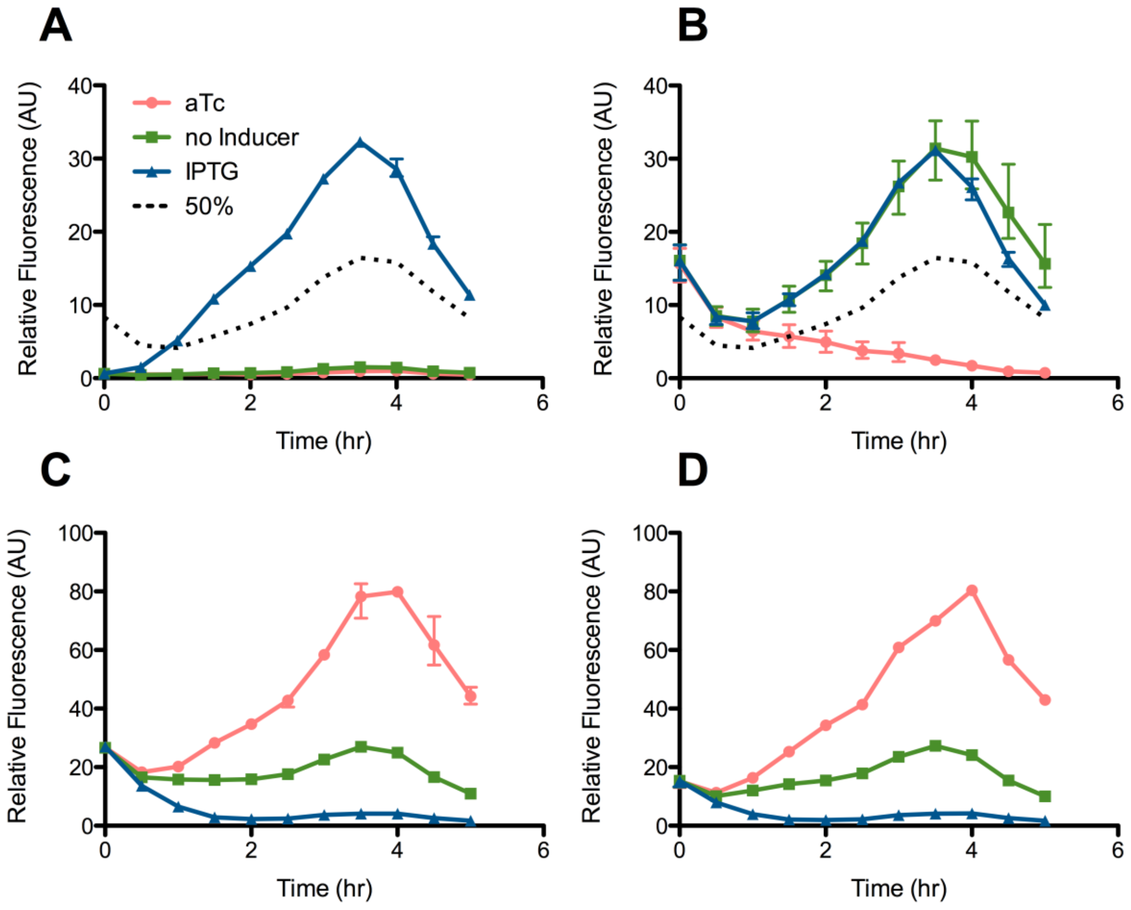


Figure S.3 - Transient switching behavior for SW5

Kinetic behavior of SW5 is shown using both types of reporters (figure S.3). (A) and (B) report using PRM-GFP. (C) and (D) report using PLtetO-1-GFP. For (A) and (C) strains are in the "on" state at time t=0. For (B) and (D) strains are initially in the "off" state. At time t=0 inducers are added into the culture to either reinforce the state, flip the state, or hold the state (no inducer added).

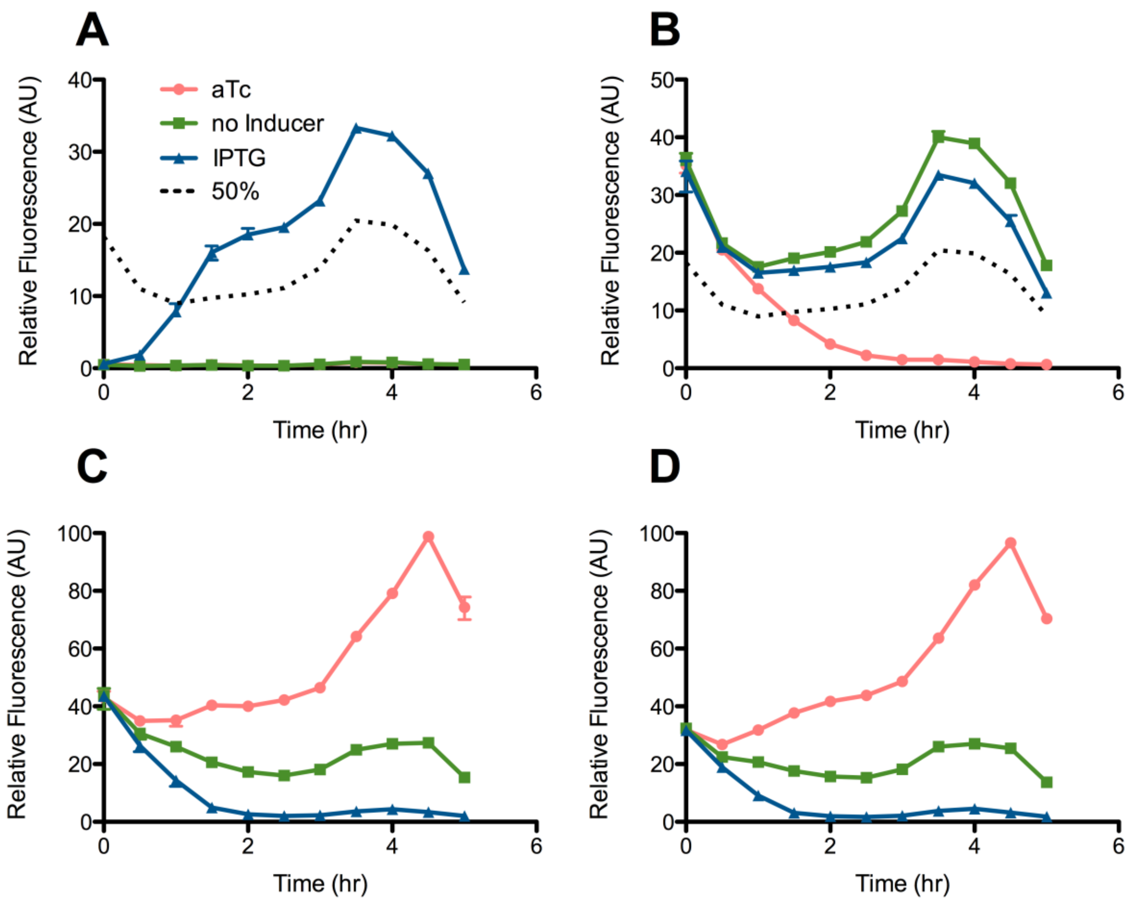


Figure S.4 - Transient switching behavior for SW6

Transient behavior of SW6 (figure S.4). The plots for SW6 are similar to figure 8 in the paper and figure S.3.

## S.3 Flow Cytometry

### S.3.1 Deactivation

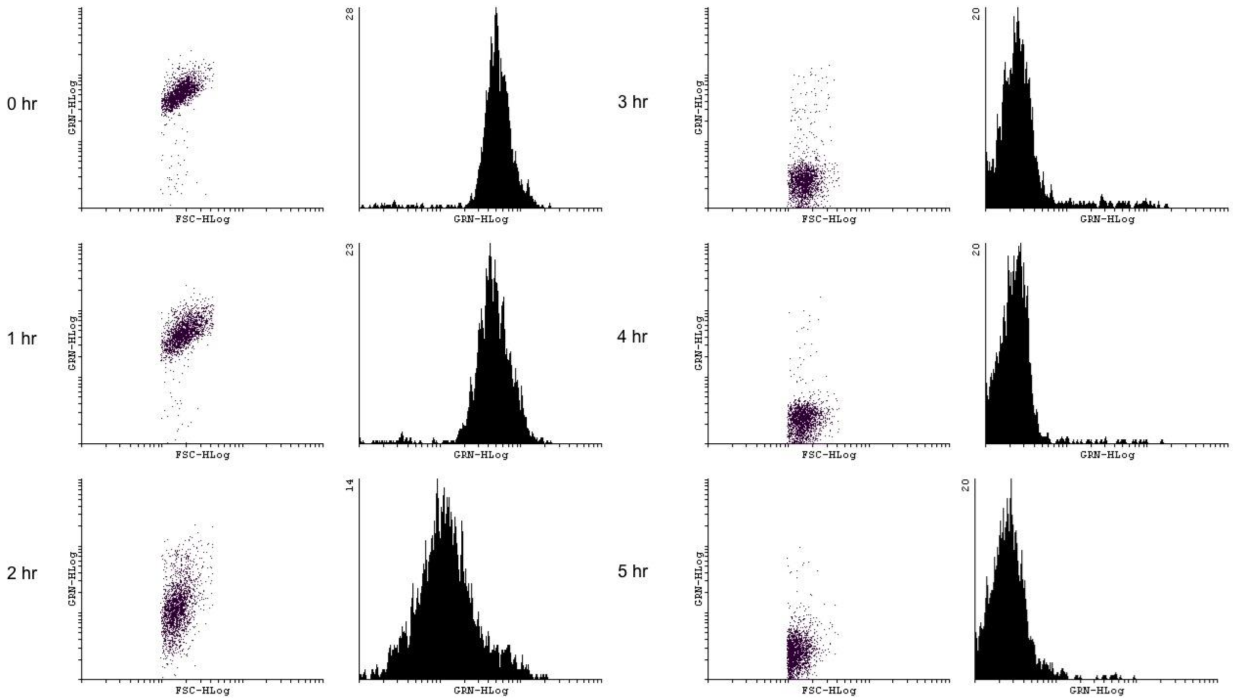


Figure S.5 – Flow cytometry of switch deactivation

This series of flow cytometry plots shows a time course for SW6 switching from the “on” to “off” state. GFP expression uses the PRM-GFP reporter. For all scatter plots, the vertical axis is GFP fluorescence measurement and the horizontal axis is the forward scattering measurement. For all histograms, the vertical axis represents the number of counts and the horizontal axis represents GFP fluorescence. During the “high” to “low” transition, usually we see a bimodal distribution representing both the “on” and “off” population. As time progresses, the “on” population shrinks and the “off” population grows. Instead of observing a bimodal distribution during the transition we see the whole “on” population move to an intermediate state, then move into the “off” state.

### S.3.2 Inducers and $P_{LtetO-1}$

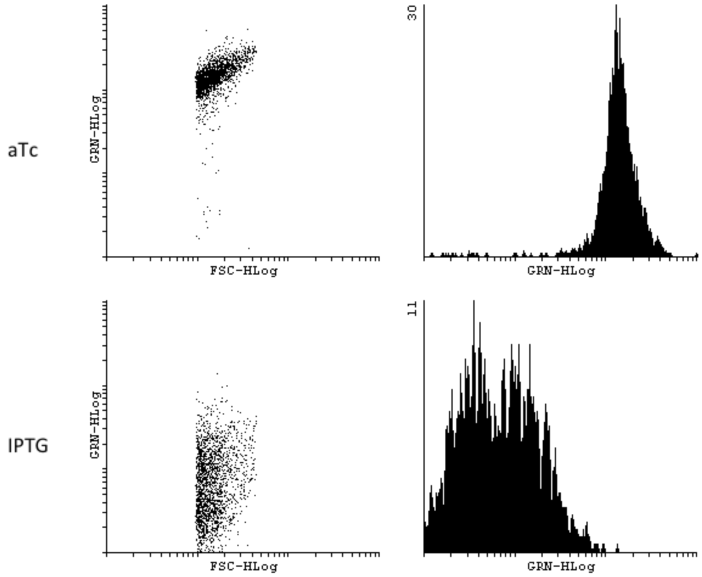


Figure S.6 – Flow cytometry with full induction by inducers

In these plots we observed the effect of adding inducers on $P_{LtetO-1}$. In the plots above GFP is reported using the PLtetO-1-GFP reporter. When aTc is added we observed high expressions of GFP shing that $P_{LtetO-1}$ is fully active (top two plots). When IPTG is added a bimodal population distribution was observed (bottom two plots). One population has no GFP expression, the other population shows that PLtetO-1 is partially active. This indicates that TetR is unable to completely shut off pLtetO-1.

### S.3.3 No inducers added

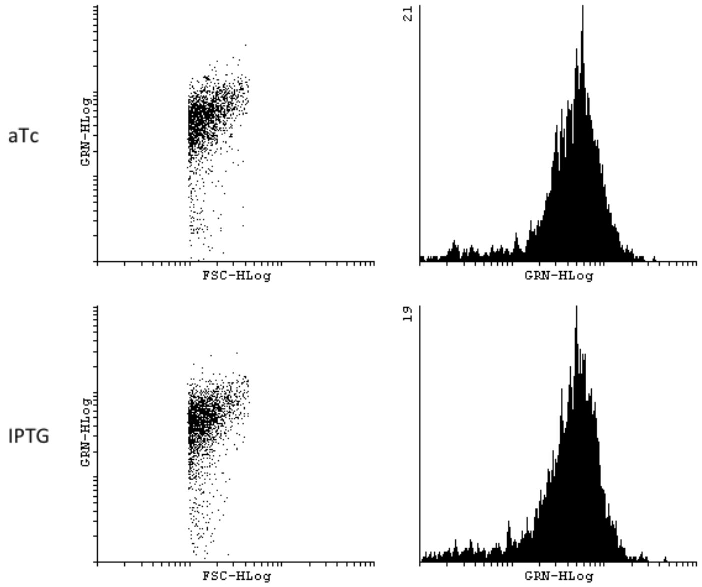


Figure S.7 – Flow cytometry without added inducers

In these plots we observed the behavior of $P_{LtetO-1}$ at steady-state. Once again the PLtetO-1-GFP reporter is used. When inducers are removed and cultures are allowed to come to steady-state a strains initially cultured overnight in aTc appear identical to strains initially cultured in IPTG.

## S.4 Single Feedback Loop Bistable System

It may be possible to create a bistable system with only the $P_{RM}$-CI positive feedback loop, and a constitutive expression of *mf*-Lon. This however would require the Michaelis constant for *mf*-Lon to be significantly smaller that the measured valued.

### S.4.1 Equations

$$\frac{d}{dt}m_{cI} = V_{P_{RM}}NC\left[\frac{1}{1+\left(\frac{K_{CI}}{p_{CI}}\right)^{n_r}} + \ell_{P_{RM}}\right] - \gamma_m m_{cI} \quad (1)$$

$$\frac{d}{dt}p_{CI} = \epsilon_{CI}m_{cI} - \gamma_{CI}p_{CI} - \frac{p_L k_{cat}}{1+\frac{K_M}{p_{CI}}} \quad (2)$$

$$p_L = constant \quad (6)$$

### S.4.2 List of parameters

| Parameters and Variables | Description | Units | Value |
|---|---|---|---|
| $m_{cI}$ | mRNA concentration for *cI* | $M$ | |
| $p_{CI}$ | Protein concentration for CI | $M$ | |
| $p_L$ | Protein concentration for *mf*-Lon | $M$ | $1\times10^{-10}$ |
| $V_{P_{RM}}$ | Velocity for mRNA production for $P_{RM}$ | $M \cdot s^{-1}$ | .3 [46] |
| $\ell_{P_{RM}}$ | mRNA leakage of $P_{RM}$ promoter | $M \cdot s^{-1}$ | 1/10 [47] |
| $\ell_{P_T}$ | mRNA leakage of $P_{LtetO-1}$ promoter | $M \cdot s^{-1}$ | 1/5050 [30] |
| $K_{CI}$ | Disassociation constant of CI to $P_{RM}$ | $M$ | $2.5\times10^{-8}$ [33] |
| $K_m$ | Michaelis constant for *mf*-Lon | $M$ | $3.7\times10^{-9}$ |
| $k_{cat}$ | Catalytic rate of *mf*-Lon | $s^{-1}$ | .071 [25] |
| $\gamma_m$ | Degradation rate of mRNA | $s^{-1}$ | $2.38\times10^{-3}$ [49] |
| $\gamma_{CI}$ | Degradation rate of CI | $s^{-1}$ | $2\times10^{-4}$ |
| $\epsilon_{CI}$ | Translation rate for CI | $s^{-1}$ | $5\times10^{-5}$ |
| $n_r$ | Hill coefficient for $P_{RM}$ | | 2[50] |
| $n_t$ | Hill coefficient for $P_{LtetO-1}$ | | 2[50] |
| $N$ | Plasmid copy number for psc101 | | 3 |
| $C$ | Concentration of a single protein/mRNA in a typical bacterium | $M$ | $1.5\times10^{-9}$[51] |

### S.4.3 Schematic

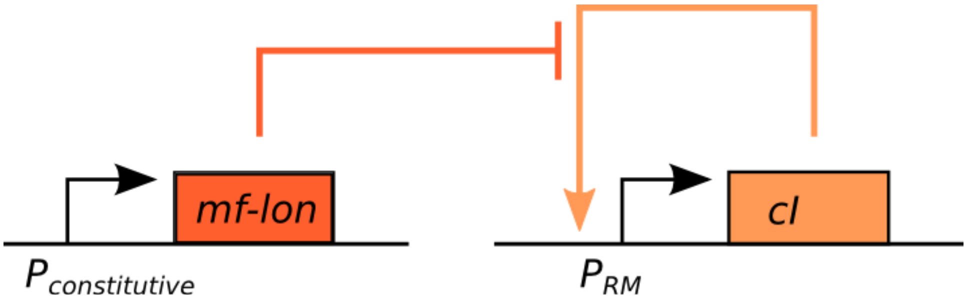


Figure S.8 – A $P_{RM}$-CI single positive feedback loop bistable circuit

### S.4.4 Rate-Balance plot

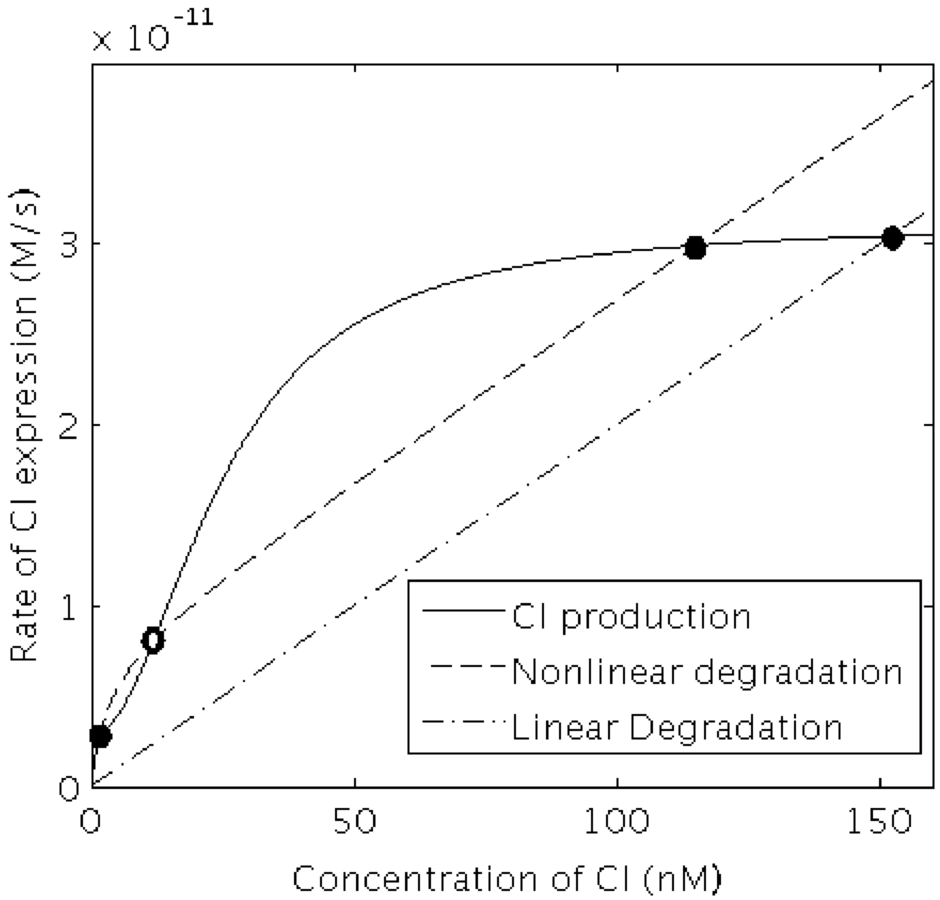


Figure S.9 – Rate-balance plot for a $P_{RM}$-CI bistable circuit

**S.5 Accession Numbers**

Accession numbers are registered with GenBank (http://www.ncbi.nlm.nih.gov/genbank/).

| Name | Accession Number |
|---|---|
| PRM-GFP | JX155239 |
| PLtetO-1-GFP | JX155236 |
| PLlacO-1-CIwt | JX155233 |
| PLlacO-1-CILVA | JX155231 |
| PLlacO-1-CImf | JX155232 |
| RFP1 | JX155240 |
| RFP2 | JX155241 |
| RFP3 | JX155242 |
| RFP4 | JX155243 |
| RFP5 | JX155244 |
| RFP6 | JX155245 |
| RFP7 | JX155246 |
| SW1 | JX155247 |
| SW2 | JX155248 |
| SW3 | JX155249 |
| SW4 | JX155250 |
| SW5 | JX155251 |
| SW6 | JX155252 |
| SW7 | JX155253 |
| PRM-CIwt-TetR | JX155238 |
| PRM-CILVA-TetR | JX155236 |
| PRM-CImf-TetR | JX155237 |
| PLlacO-1-mfLon | JX155234 |
